# Pre-partum feeding strategies affect colostrum metabolite levels related to nitrogen and energy metabolism in Holstein dairy cows

**DOI:** 10.1007/s11306-025-02329-w

**Published:** 2025-08-29

**Authors:** Paraskevi Tsermoula, Niels Bastian Kristensen, Bekzod Khakimov

**Affiliations:** 1 Department of Food Science, University of Copenhagen, Rolighedsvej 26, Frederiksberg, 1958 Denmark; 2 SEGES Innovation P/S, Agro Food Park 15, Aarhus N, DK 8200 Denmark; 3 Present Address: Department of Animal and Veterinary Sciences, Aarhus University Viborg, AU-Foulum, Tjele, 8830 Denmark

**Keywords:** Colostrum, Metabolome, Prepartum feeding, Periparturient cows

## Abstract

**Introduction:**

Cow colostrum synthesis takes place during the last month of pregnancy. Its composition is influenced by individual and environmental factors, such as cow parity, feeding, season and environmental conditions. Therefore, colostrum metabolomic profiling may provide information about the physiological status of cows around calving.

**Objectives:**

The cow colostrum metabolome was analyzed to determine whether its variability could be used to elucidate the cows’ physiological status around calving and provide insights into the outcomes of cow transition programs.

**Methods:**

The factors assessed included a control feeding based on grass-clover silage and barley straw (FAR), two phase feedings based on acidified corn silage and canola cake, supplemented with magnesium chloride (MGC) or magnesium chloride and ammonium chloride (NH_4_) and a feeding consisting of one week of grass-diluted MGC followed by two weeks of the NH_4_. Colostrum was collected from 89 dairy cows, which were randomly allocated to the feedings three weeks before the expected calving date during spring, summer and autumn. Cow colostrum samples were analyzed using proton nuclear magnetic resonance spectroscopy.

**Results:**

Our results show that calving season influenced the levels of 14 metabolites. Independent of seasonal variation, acidified corn silage diets resulted in consistent decreased levels of tryptophan, acetate and cytidine, while the non-acidified grass-based diet resulted in increased concentrations of fucose.

**Conclusions:**

Although colostrum is physiologically regulated, our findings, for the first time, indicate that the four feeding strategies induce shifts in fucose, tryptophan, acetate and cytidine levels, reflecting the energy and nitrogen metabolism of cows before parturition.

**Supplementary Information:**

The online version contains supplementary material available at 10.1007/s11306-025-02329-w.

## Introduction

Colostrum is defined as the mammary secretions collected during the first milking postpartum and serves as a source of immunoglobulins and nutrients for the newborn (McGrath et al., 2016). Lactose, fatty acid synthesis and other enzymatic processes begin two to four weeks before parturition, promoting the accumulation of nutrients in the mammary gland (Hartmann, 1973; Mellenberger et al., 2009). Colostrum composition in lactating cows exhibits both individual (breed, parity, gestational length, calf sex, udder and cow health) and environmental (feeding, season, environmental conditions, time to harvest, dry-period length, time in Close-Up pen) variability (Westhoff et al., 2024). Although season, prepartum feeding strategies, and individual factors have been shown to influence the colostrum metabolome (O’Callaghan et al., 2021), their interactions and impact on markers of cows’ physiological status require further investigation.

Colostrum synthesis overlaps with the transition period, one of the most challenging stages of their production cycle. This is due to the rapid increase in nutrient demands required to support fetal growth, as well as colostrum and milk production (Bell, 1995). Due to the physiological importance of this period, research has focused on investigating the effect of prepartum feeding, season, environment and cow individual factors on the yield, protein content and immunological quality of colostrum (Soufleri et al., 2021). However, only one study has analyzed the colostrum metabolome as a function of feeding, season and parity. O’Callaghan et al. (2021) examined the metabolome of cow colostrum and milk during the first five days of lactation. Although this study demonstrated that parity did not show to significantly affect colostrum metabolome, the effect of other factors including dry-period length and prepartum feeding on colostrum metabolome may influence early-life microbial colonization and calf development and thus require further investigation.

The colostrum samples of the present study have been utilized by Tsermoula et al. (2024) for analytical method development purposes, highlighting that ^1^H NMR spectroscopy combined with SigMa (Signature Mapping) software is an efficient approach for identifying and quantifying metabolites in colostrum. To the authors’ knowledge, no study has investigated the combined effects of different feeding strategies and seasonality on the colostrum metabolome. Toward this direction, this study examines the variability of colostrum metabolites, highlighting the impact of different feeding strategies across spring, summer and autumn, aiming to elucidate the cows’ physiological status around calving. The study also discusses potential biomarkers (fucose, acetate, tryptophan and cytidine), which can provide new insights into the metabolism of cows around calving.

## Materials and methods

### Experimental design

**Fig. 1:**
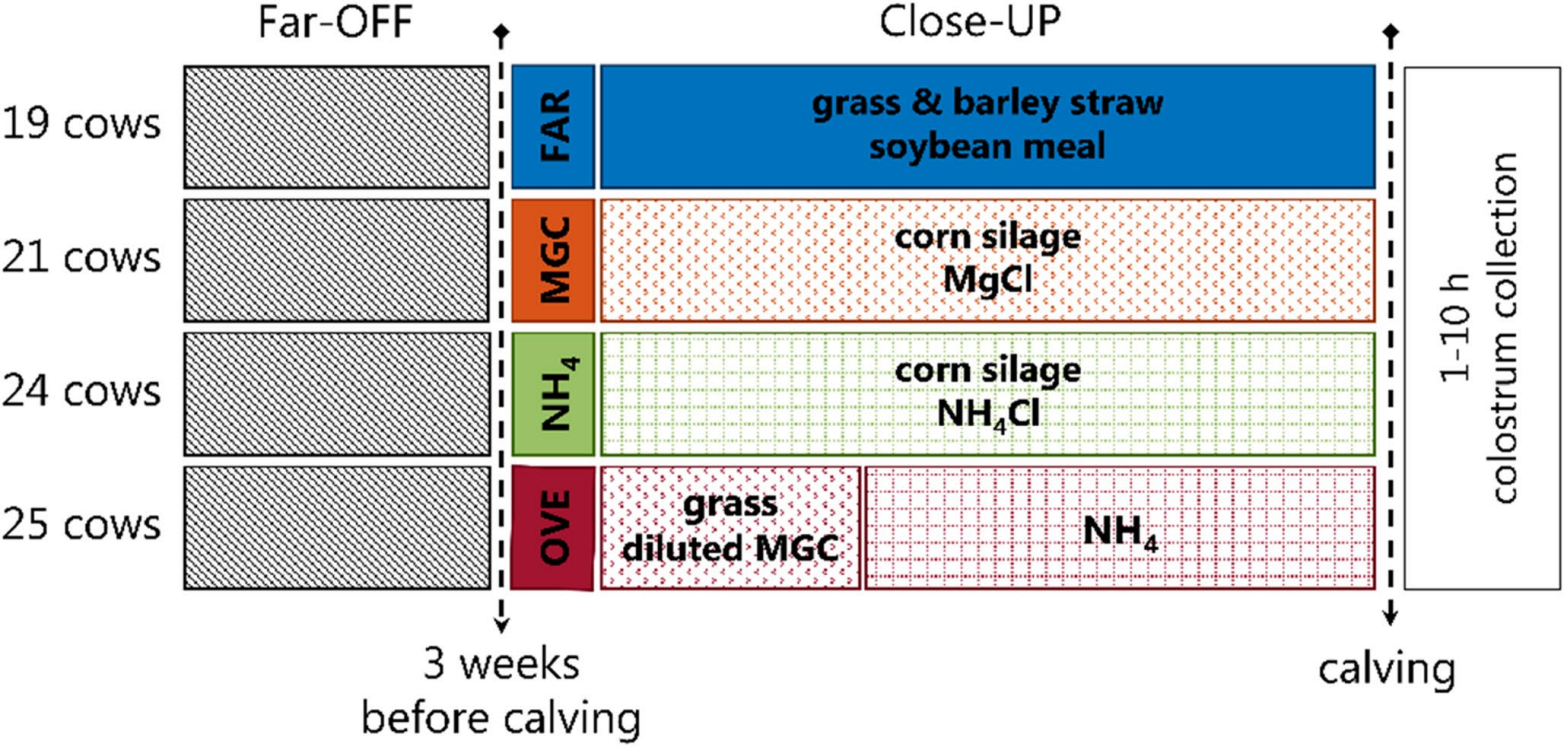
Overview of experimental design and cow feeding strategies. A total of 89 cows followed a grass and barley straw-based ration with soybean meal diet until three weeks before calving (i.e. Far-OFF period). In sequence, the cows were allocated to the following four different feedings over the three weeks of Close-Up period; 19 cows were allocated to FAR feeding, a grass-based ration with soybean meal, 21 cows to MGC feeding, a corn silage-based ration acidified with magnesium chloride, 24 cows to NH_4_ feeding, a corn silage-based ration acidified with magnesium chloride and ammonium chloride and 25 cows to OVE feeding, a diluted grass MGC ration for one week and the NH_4_ ration for two weeks. Colostrum samples were collected within ten hours after parturition

A total of 39 second parity (i.e. primiparous cows during the dry period before their second calving) and 50 multiparous Danish Holstein cows were randomly allocated to four feeding treatments during their dry period at the Danish Cattle Research Centre (Department of Animal and Veterinary Sciences at Aarhus University, Tjele, Denmark). Throughout the Far-OFF period (early dry period until three weeks before calving), all cows received a standardized Far-OFF ration (grass and barley straw-based ration formulated to achieve an organic matter digestibility of 65%, with soybean meal to maintain a crude protein concentration of at least 110 g/kg dry matter (DM)). During the Close-Up period (three weeks before expected calving), cows were randomly assigned to four feeding strategies, as shown in Fig. 1.

During the Close-Up period, the control ration FAR was a low-energy non-acidified, grass silage- and barley straw-based Far-OFF ration with + 299 mEq/kg DM dietary cation-anion difference (DCAD) from dry-off to calving. The remaining three rations followed phase feeding, where cows received a grass-based Far-OFF ration followed by different Close-UP rations three weeks before expected calving. The MGC ration included corn silage and canola cake acidified with MgCl₂ and was formulated to provide 200 g/kg DM energy, at least 130 g/kg DM protein and − 39 mEq/kg DM DCAD to support prepartum metabolic adaptation. The NH₄ ration was a MGC ration with added ammonium chloride to further reduce DCAD to -96 mEq/kg DM. Lastly, the OVE group received a neutral transition grass diluted (20:80 on a DM basis) MGC diet during the first Close-UP week followed by the NH₄ diet until calving with an overall DCAD of -4 mEq/kg DM, aiming to ease the transition between Far-OFF and Close-UP rations.

Calving took place between February and October of 2021. From the FAR-fed cows 7 calved in spring, 6 in summer and 6 in autumn. From the MGC-fed cows 6 calved in spring, 8 in summer and 7 in autumn. From the NH_4_-fed cows 5 calved in spring, 9 in summer and 10 in autumn and from the OVE-fed cows 7 calved in spring, 10 in summer and 8 in autumn.

### Housing and management

Cows entered the dry-off pen 56 days before expected calving and they were receiving the Far-OFF ration. During the dry-off and the far-off periods, cows were housed in freestalls with slated floors and bedded with mattresses and wood shavings. Feed was offered in gated weighing feed stations (Hokofarm Group, Emmeloord, The Netherlands). During the Far-OFF period, all cows had access to all feeding stations with 2 cows per feeding station. Cows fed the FAR ration during the Close-Up period were moved to straw bedded calving pens approximately one week before calving. The cows fed the OVE ration, were moved to a separate freestall pen during the Close-Up period. The cows fed the MGC and NH_4_ rations were housed in a deep bedding pen during the Close-UP period. All Close-UP feedings were offered in gated weighing stations with restricted access. All feeding stations were validated weekly using a 10-kg test weight.

### Sample collection

Colostrum samples were collected from cows in the calving pen within 10 h from calving, using a portable milking machine, between February 28, 2021, and October 14, 2021. The average time from calving to colostrum collection was 2.6, 3.9, 2 and 2.1 h for the FAR, MGC, NH_4_ and OVE-fed groups, respectively. Cows were milked into individual polycarbonate buckets and after colostrum was mixed manually, a sample was collected in a 100 mL screw cap cup (Sarstedt AG & Co. KG, Nümbrecht, Germany), immediately stored at − 20 °C on-farm, then transferred to − 80 °C upon arrival at the laboratory, where they remained until analysis.

### Proximate composition

Ingredients and total mixed rations (TMR) were sampled weekly, stored at -20 °C and combined monthly for nutrient analysis. Samples were dried at 60 °C for 48 h in a forced-air oven for weekly DM determination. The rations composition (crude protein, crude fat, neutral detergent fiber, starch) were analyzed by Fourier Transform-Near Infrared (FT-NIR) spectroscopy (Bruker MPA, Bruker Optics GmbH, Ettlingen, Germany) at Kvægbrugets Forsøgslaboratorium (KFL), Skejby, Denmark. The TMR samples were analyzed for mineral composition using inductively coupled plasma mass spectrometry (ICP-MS) and chloride (Cl) was analyzed using silver nitrate titration, at a commercial laboratory (Eurofins Agro Testing Denmark A/S). Colostrum samples were analyzed for fat and protein composition by Fourier Transform-Near Infrared (FT-NIR) spectroscopy (Bruker MPA, Bruker Optics GmbH, Ettlingen, Germany) at Kvægbrugets Forsøgslaboratorium (KFL), Skejby, Denmark and for Brix by light scattering using a digital refractometer (PAL-1, Atago, Tokyo, Japan).

### Feed intake monitoring

Feed intake was calculated for each cow on a 24 h basis, starting at 10:00 am. Dry matter intake (DMI) for the Close-Up period and one day before calving (prefresh period) was calculated by multiplying the weight of the consumed feed as measured by the weighing stations with the weekly mean DM of feed rations as measured using the forced-air oven. Energy and protein values in TMR and net energy for lactation (NEL20) at 20 kg DMI/day, were calculated using the Nordic Feed Evaluation System (NorFor).

### Sample Preparation for ^1^H NMR analysis

Colostrum samples were thawed at 40 °C for 30 min and diluted in a 1:4 ratio (v: v) with ultrapure water from a Millipore lab water system (Merck KGaA, Darmstadt, Germany), equipped with a 0.22 μm filter membrane. Diluted colostrum was defatted by centrifuging at 1,500 × g for 30 min at 4 °C and 550 µL of skimmed colostrum was transferred in a 10 kDa cutoff spin-filter (Amicon Ultra-0.5 Centrifugal Filter Unit, Sigma-Aldrich, Søborg, Denmark) and centrifuged at 21,000 × g for 30 min at 20 °C to remove proteins. Then, 400 µL ultrafiltered colostrum were mixed with 200 µL of 0.2 M potassium phosphate buffer in D_2_O (containing 1 mg/mL TSP (trimethylsilylpropanoic acid), 3 mg/mL gallic acid, and 0.13 mg/mL NaN_3_ at pH 6.0), vigorously vortexed for 10 s, and 550 µL was transferred into NMR SampleJet tubes of L = 103.5 mm and O.D. = 5.0 mm (Bruker Biospin, Ettlingen, Germany) before NMR analysis.

### ^1^H NMR spectral acquisition and data processing

^1^H NMR spectra were recorded using a Bruker Advance III 600 MHz NMR spectrometer equipped with a 5 mm broadband inverse probe, automated tuning and matching accessory (ATMA), cooling unit (BCU-05), and an automated sample changer (Sample Jet, Bruker Biospin, Rheinstetten, Germany) with sample cooling (278 K) and preheating stations (298 K). Data acquisition and processing were carried out using TOPSPIN 3.5 (Bruker Biospin, Rheinstetten, Germany) and automation of the measurements was controlled by IconNMR (Bruker Biospin, Rheinstetten, Germany). Before measurement, samples were preheated for 300 s in the SampleJet and equilibrated at 300 ± 0.1 K for 3 min inside the probe head. Automatic tuning and matching, lock, and shimming (TOPSHIM routine) were performed for each sample. The ^1^H NMR spectra were acquired using the pulse sequence with water suppression, *noesygppr1d*, from the Bruker pulse library. 32 scans were acquired after 4 dummy scans, and the generated free induction decays (FIDs) were collected into 65.5k data points using a spectral width of 20 ppm. Recycle delay, mixing time and receiver gain were set to 4.0 s, 0.01 s, and 16 respectively. Spectra were automatically phased and baseline corrected in TOPSPIN software. Prior to Fourier transform, a line broadening function of 0.3 Hz was applied. ^1^H NMR spectra were imported into the SigMa software (Khakimov et al., 2020) and aligned towards the TSP singlet at 0 ppm using *icoshift* (Savorani et al., 2010). Metabolite annotation was performed using SigMa, which utilizes a chemical shift library including 63 common milk metabolites. This library was developed by acquiring spectra of authentic standards and spectra of raw milk samples spiked with the same standards as detailed by Tsermoula et al. (2024). In the case of orotic acid and ethanol, annotation was performed using literature data (Sundekilde et al., 2014).

Quantification of metabolites was performed by integrating and correcting gallic acid signal at 7.2 ppm according to the two aryl protons and comparing to the integrals of the metabolites as described by Tsermoula et al. (2024). In brief, the absolute concentration in molarity of each metabolite was calculated as follows for Eq. 1:

1 $$\:{C}_{x\:}=\:\frac{{C}_{gal}\:\times\:\:{N}_{gal}\:\times\:\:{MW}_{x}\:\times\:\:{I}_{x}}{{N}_{x}\:\times\:\:{I}_{gal}\:\times\:\:{MW}_{gal}}$$

where C, N, MW, and I are the concentration, number of nuclei, molecular weight, and integral area of the compounds of interest (x) and gallic acid (gal), respectively.

### Statistical analysis

Differences in colostrum yield, DM, fat and protein content, and DMI, NEL20 and crude protein intake of cows across feeding groups and differences in colostrum metabolites between the feeding strategies and seasonality were evaluated using one-way analysis of variance (ANOVA) including Benjamini–Hochberg’s false discovery rate (FDR) correction (5%). Multiple linear regression (MLR) analysis was used to test the effect of feedings on colostrum metabolites. Separate models were developed for each metabolite, with metabolites as the dependent variable and feedings as the independent variable (categorical variable; class 1 = FAR, 2 = MGC, 3 = NH_4_, 4 = OVE) adjusted for DMI in close up period (continuous variable), DMI in prefresh period (continuous variable), time from calving to colostrum harvest (continuous variable), season (categorical variable, 1 = spring (February until April), 2 = summer (May until July), 3 = autumn (August until October) and Brix (continuous variable). Confounders were evaluated for association with the feeding treatments using independent t-tests for continuous variables and chi-square tests for categorical variables. ANOVA-simultaneous component analysis (ASCA) (Smilde et al., 2005) was performed to explore the data. Metabolites levels in mM are reported as means ± standard error unless otherwise stated. Data analysis was performed in MATLAB using the PLS_Toolbox 8.9.2 (Eigenvector Research Inc., Manson, WA USA). 

## Results

### Colostrum parameters and feed intake

**Fig. 2:**
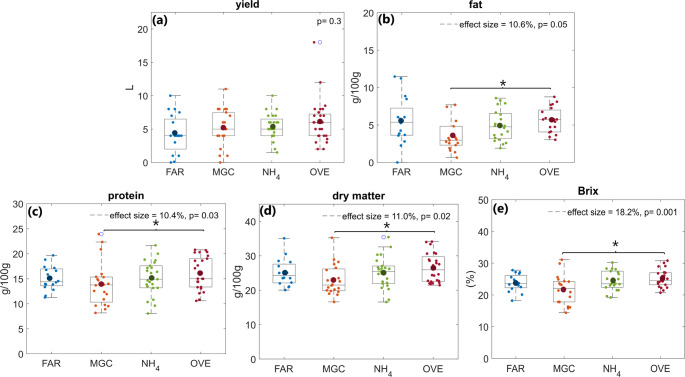
Box plots showing the (**a**) yield (L), (**b**) Brix (%), (**c**) fat content (%), (**d**) protein content (%) and dry matter (DM) content (%) of colostrum as sampled from the FAR, MGC, NH4 and OVE-fed cows. The horizontal line and bold dot inside the boxes represent median and mean values, respectively. FDR corrected p-values and effect sizes (%) were calculated using ANOVA. *p-value ≤ 0.05 represents significant difference between feedings

Colostrum parameters including the yield, DM, fat and protein content, and Brix are shown in Fig. 2. Colostrum yield was 4.4 ± 0.7 L, 5.2 ± 0.6 L, 5.4 ± 0.4 L and 6.1 ± 0.7 L for the FAR, MGC, NH_4_ and OVE-fed cows, respectively, with feeding strategies having no significant effect (p-value = 0.3; Fig. 2a). The fat, protein and DM contents were significantly lower (p-value ≤ 0.05) in the MGC group (fat 4.9 ± 0.7%, protein 13.3 ± 1.1%, DM 22.0 ± 1.3%) compared to the OVE group (fat 6.3 ± 0.6%, protein 16.6 ± 0.8%, DM 26.7 ± 1.0%). In contrast, the FAR group (fat 6.0 ± 0.9%, protein 13.8 ± 1.1%, DM 23.5 ± 1.6%) and the NH_4_ group (fat 5.4 ± 0.6%, protein 14.5 ± 0.9%, DM 24.0 ± 1.4%) showed intermediate values (Fig. 2b, c and d). Cows fed the FAR, NH_4_ and OVE diets produced colostrum with similar Brix (22.6 ± 1.5%, 25.4 ± 1.1% and 25.7 ± 0.8%, respectively), which were above 20% in all cases. Cows fed with the MGC diet produced colostrum with 20.7 ± 1.4% Brix, which was significantly lower (p-value = 0.001) than that of cows fed the OVE diet (Fig. 2e).

The DMI, NEL20 and crude protein (cp) intake of the FAR-fed cows during both the Close-Up (DMI 11.5 ± 0.3 Kg/day, NEL20 53.5 ± 1.3 MJ/day, cp. 1,376 ± 55 g/day) and prefresh (DMI 9.2 ± 0.3 Kg/day, NEL20 43.0 ± 1.7 MJ/day, cp. 1,100 ± 54 g/day) periods were significantly lower (p-value < 0.05) than those of the MGC, NH_4_ and OVE–fed cows (Fig. S1). The MGC, NH_4_ and OVE–fed cows did not show any significant differences in intake parameters during either the Close-Up or prefresh periods. During the Close-Up period, DMI was 16.3 ± 1.0, 16.0 ± 1.2 and 15.9 ± 0.7 kg/day; NEL20 was 104 ± 6.5, 102 ± 7.7, and 101 ± 4.9 MJ/day; and crude protein intake was 2,199 ± 130, 2,194 ± 158, and 2,173 ± 111 g/day for the MGC, NH₄, and OVE groups, respectively. In the prefresh period, DMI was 12.7 ± 0.5, 13.6 ± 1.2, and 12.2 ± 0.8 kg/day; NEL20 intake was 80.7 ± 3.4, 86.9 ± 7.5, and 76.7 ± 5.2 MJ/day; and crude protein intake was 1,718 ± 71, 1,860 ± 157, and 1,653 ± 114 g/day for MGC, NH₄, and OVE, respectively.

### Colostrum metabolome

In total, 55 metabolites were identified and 47 of them were quantified in colostrum. The quantified compounds include 10 amino acids, 5 amino acid derivatives, 13 organic acids, 6 sugars and their derivatives, 3 amino alcohols, 3 nucleotide/sides and 7 compounds including pantothenic acid, butyric acid, ethanol, acetone, trimethylamine, dimethyl sulfone and methanol. The 8 non-quantified compounds include fatty and amino acids however, their pure concentration remained unknown due to overlapping signals. Table 1 shows the average concentration of each of the metabolites in colostrum from 89 cows.

**Table 1:** Concentration (mM) of the 47 quantified metabolites in 89 colostrum samples from first milking, expressed as average ± standard deviation

Metabolite	Chemical shift	Multiplicity	Concentration (mM)
Pantothenic acid	0.94	s	0.043 ± 0.042
Leucine^a, b^	0.96	d	0.113 ± 0.102
Valine^ab^	0.99	d	0.158 ± 0.116
Isoleucine^a, b^	1.02	t	0.071 ± 0.066
Ethanol	1.19	t	0.045 ± 0.012
Fucose^a, b^	1.25	d	0.022 ± 0.018
Lactic acid^a, b^	1.33	d	0.057 ± 0.041
Alanine^a, b^	1.48	d	0.097 ± 0.083
Butyric acid	1.55	q	0.243 ± 0.110
Acetic acid^a, b^	1.92	s	0.136 ± 0.045
n-Acetyl glucosamine^b^	2.06	s	0.294 ± 0.172
Acetylcholine	2.15	s	0.075 ± 0.030
Acetone^a, b^	2.23	s	0.038 ± 0.020
Acetoacetic acid	2.28	s	0 079 ± 0.048
Glutamic acid^b^	2.30	m	0.014 ± 0.07
Glutamine	2.36	t	0.072 ± 0.049
Pyruvic acid^a, b^	2.38	s	0.042 ± 0.040
Succinic acid^a, b^	2.41	s	0.056 ± 0.016
Citric acid^a, b^	2.57	d	4.340 ± 1.502
Aspartic acid	2.77	d	0.573 ± 0.182
Trimethylamine^b^	2.89	s	0.053 ± 0.011
Creatine^a, b^	3.04	s	0.649 ± 0.288
Creatinine^a, b^	3.05	s	0.272 ± 0.141
Malonic acid^b^	3.11	s	0.166 ± 0.081
Dimethyl sulfone	3.15	s	0.132 ± 0.074
Acetylcarnitine^a, b^	3.19	s	0.325 ± 0.116
Choline^a, b^	3.20	s	0.244 ± 0.125
Carnitine^a, b^	3.23	s	0.403 ± 0.101
Betaine^a, b^	3.26	s	0.376 ± 0.137
Methanol	3.34	s	0.067 ± 0.023
Phosphocholine^b^	4.33	m	1.611 ± 0.023
α-Lactose^a, b^	5.24	d	21.20 ± 5.253
Galactose	5.27	d	0.179 ± 0.108
Glucose 1-phosphate^a, b^	5.42	q	0.370 ± 0.313
Galactose 1-phosphate^a, b^	5.50	dd	0.851 ± 0.320
Uridine^a, b^	5.91	d	0.621 ± 0.419
Cytidine	6.07	d	0.044 ± 0.019
Orotic acid^a^	6.20	s	0.026 ± 0.011
Fumaric acid^b^	6.52	s	0.019 ± 0.008
Cis-aconitic acid^b^	6.59	s	0.007 ± 0.006
Tyrosine	6.91	m	0.030 ± 0.022
Phenylalanine^b^	7.34	m	0.021 ± 0.013
Uracil	7.55	d	0.099 ± 0.071
Tryptophan	7.56	d	0.143 ± 0.043
Hippuric acid^a, b^	7.64	m	0.074 ± 0.023
Histidine	7.93	s	0.072 ± 0.009
Formic acid^b^	8.46	s	0.037 ± 0.027

Metabolites previously identified and quantified in cow colostrum by ^a^O’Callaghan et al. (2021) and ^b^Amarjeet et al. (2025).

Previously we discovered that the FAR, MGC, NH_4_ and OVE feedings have a marginally significant effect (4.5% variation, p-value = 0.1) on colostrum metabolome (Tsermoula et al., 2024). In the present study, cow parity and calving season were included in the ASCA model and showed that both factors significantly affect the colostrum metabolome (p-value ≤ 0.05), introducing 2.7% and 7.5% of variation, respectively.

### Variations in colostrum metabolome from cows following different feedings

One-way ANOVA revealed that the concentrations of 21 metabolites were significantly different (p-value ≤ 0.05, FDR = 5%) across the four feeding strategies (Table 2). However, DMI in the Close-Up and prefresh period, time from calving to milking, season and Brix confounded with the feeding strategies, as determined by chi-square or t-test analysis (p-value < 0.05). To evaluate their possible interference, a MLR model was built, where the confounding factors were treated as fixed effects while testing the feeding effect. Four metabolites including fucose, tryptophan, acetic acid and cytidine, remained significant for the feeding factor (p-value < 0.05) (Table 2).

**Table 2:** List of metabolites that differ in their concentrations in colostrum from 89 cows under the four different feeding strategies found in ANOVA and multiple linear regression analyses

	*p*-value^a^	effect (%)		*p*-value^b^					
				feeding	DMI^c^ Close-Up	DMI^a^ prefresh	time to first milking	season	Brix
pantothenate	0.02	12.5		–	–	–	–	–	0.03
valine	0.01	13.1		–	–	–	–	–	0.04
isoleucine	0.03	11.3		–	–	–	–	–	0.001
fucose	0.03	12.7		0.02	–	–	–	0.001	–
alanine	0.03	11.5		–	–	–	–	0.01	–
acetic acid	0.05	10.2		0.02	–	0.01	–	–	–
glutamate	0.01	14.3		–	–	–	–	0.01	–
succinate	0.02	12.5		–	–	–	–	–	–
pyruvate	0.001	25.5		–	–	–	–	0.03	–
citrate	0.04	11.6		–	–	–	0.01	–	–
dimethylamine	0.01	15.4		–	–	–	–	–	–
creatinine	0.001	19.2		–	–	–	–	–	–
dimethyl sulfone	0.001	53.1		–	–	–	–	–	–
choline	0.02	15.5		–	–	0.005	–	–	0.001
galactose	0.003	23.8		–	–	0.003	–	–	0.02
glucose-1-phosphate	0.01	15.1		–	–	–	0.04	–	–
uridine	0.002	19.4		–	–	–	–	–	0.001
cytidine	0.03	12.7		0.04	–	–	–	–	–
uracil	0.04	9.75		–	–	–	–	–	–
tryptophan	0.02	11.2		0.04	0.001	0.03	–	–	0.02
hippurate	0.02	13.4		–	0.01	–	–	–	0.02

^a^ANOVA p-values between the feeding groups are false discovery rate corrected (FDR, 5%)

^b^MLR p-values

^c^DMI: dry matter intake

**Fig. 3:**
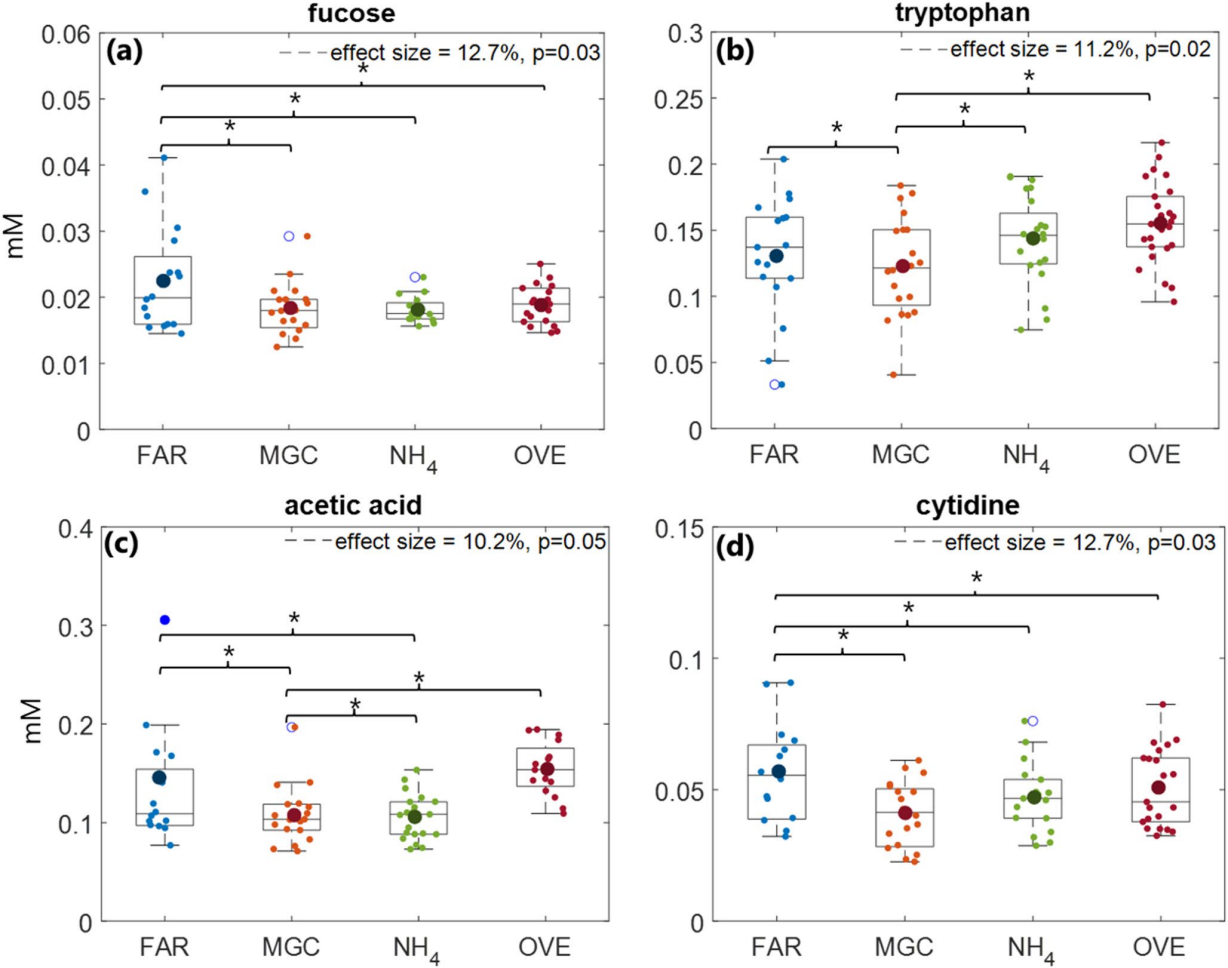
Variation of **a)** fucose, **b)** tryptophan, **c)** acetic acid and **d)** cytidine among FAR, MGC, NH_4_ and OVE colostrum samples of the cows under different feedings. Box plots show the absolute concentration of the compounds, FDR corrected p-values and effect size (%) are calculated from ANOVA performed on the absolute concentrations (mM)

Figure 3 shows the variability distribution for fucose tryptophan, acetic acid and cytidine in box plots. Fucose was significantly decreased in colostrum from FAR-fed cows (0.023 ± 0.002 mM), while it was found in similar levels in the rest of MGC, NH_4_ and OVE samples (0.021 ± 0.002 mM, 0.019 ± 0.001 mM and 0.019 ± 0.001 mM, respectively) (Fig. 3a). Tryptophan concentration was similar in OVE (0.149 ± 0.007 mM), FAR (0.141 ± 0.010 mM) and NH_4_ (0.144 ± 0.009 mM) colostrum but significantly lower in MGC (0.131 ± 0.011 mM) (Fig. 3b). Acetic acid concentration followed the trend MGC, NH_4_ < OVE, FAR (0.088 ± 0.008, 0.088 ± 0.008 < 0.136 ± 0.007, 0.146 ± 0.016 mM; Fig. 3c) and as is shown in Table 2 its concentration was also affected by the DMI in the prefresh period (p-value = 0.01). Finally, cytidine concentration was higher in the FAR (0.051 ± 0.006 mM) compared to MGC (0.040 ± 0.003 mM), NH_4_ (0.042 ± 0.004 mM), and OVE (0.046 ± 0.003 mM) (p-value = 0.03), with no significant differences in MGC, NH_4_, and OVE concentrations (Fig. 3d).

### Effect of calving season on colostrum metabolome

**Fig. 4:**
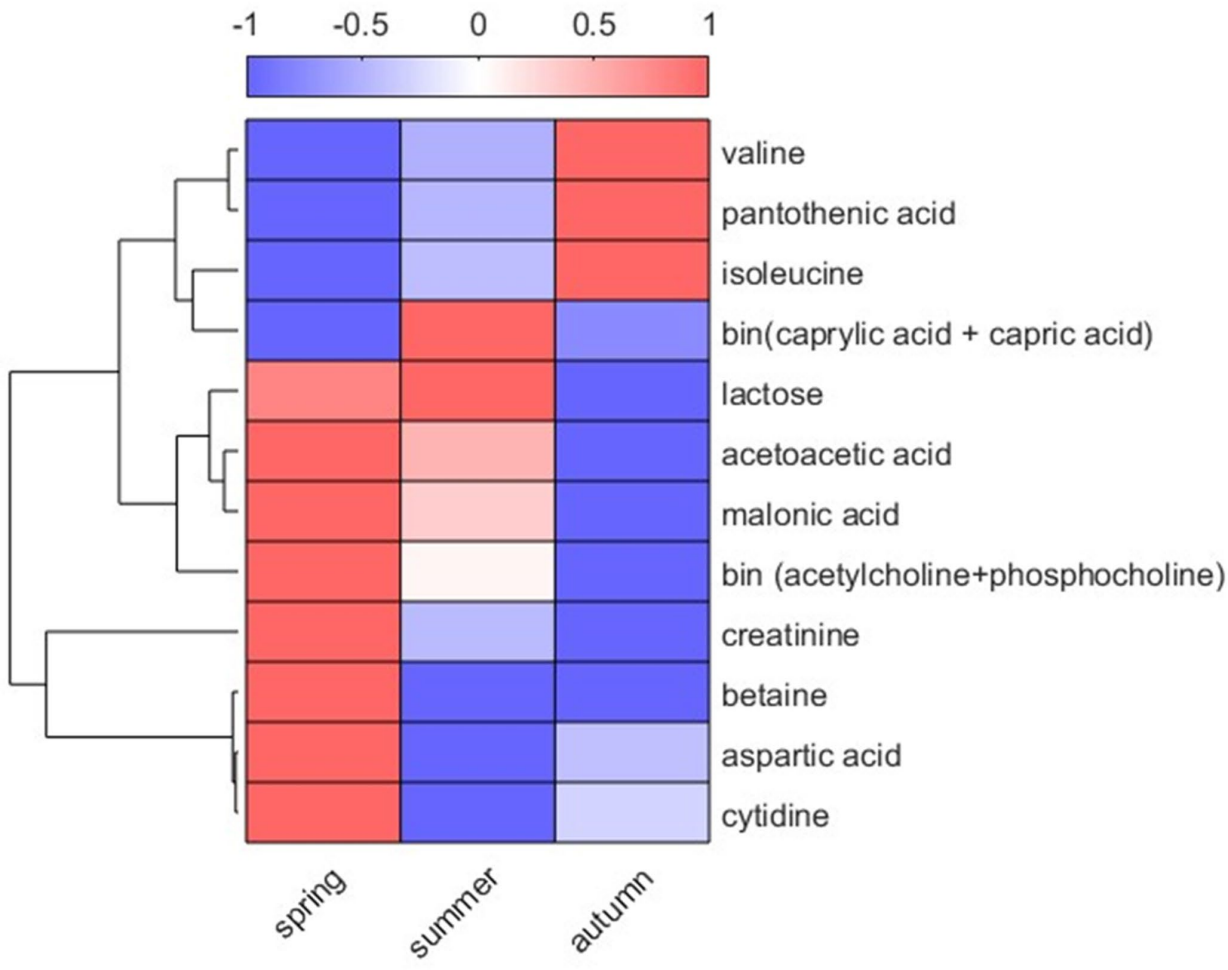
Heatmap showing the variation in the levels of 14 metabolites across seasons (spring, summer, autumn). Metabolites were selected based on their significant seasonal variation using ANOVA with FDR corrected p-value < 0.05. The intensity of the colors represents the normalized relative mean abundance of each metabolite across all cows per season. The normalized data are presented between − 1 and 1, where 1 (red color) indicates higher metabolite levels, zero (white color) indicates intermediate metabolite levels, while − 1 (blue color) represents the lowest relative mean abundance. Hierarchical clustering of metabolites was performed using Euclidean distance and average linkage, the dendrogram is shown on the left

ANOVA-Simultaneous component analysis showed that calving season accounts for the biggest variation in the colostrum metabolome (7.5%), when compared to the feeding strategies and parity (variation 4.5% and 2.7%, respectively). The effect of calving season on the colostrum metabolome was evaluated with one-way ANOVA analysis (see materials and methods). The analysis revealed that 14 metabolites were significantly different between the 3 seasons (Table S2; Fig. 4). Betaine, creatinine, aspartic acid, cytidine, acetylcholine, phosphocholine, malonate, acetoacetate, and lactose levels consistently decreased (p-value < 0.05), whereas, valine, pantothenate and isoleucine levels increased (p-value < 0.05) from spring to summer and autumn. When comparing changes in metabolites between spring and summer, increased levels were observed for caprylic and capric acid (p-value < 0.05), whereas acetoacetate, aspartic acid, betaine and cytidine levels decreased (p-value < 0.05). Finally, pantothenate and valine levels increased (p-value < 0.05) and acetoacetate, lactose, capric and caprylic acid levels decreased in summer compared to autumn (p-value < 0.05).

## Discussion

The transition period in dairy cows is defined as the 3 weeks pre-calving until 3 weeks post-calving and is characterized by physiological, metabolic and nutritional changes. Colostrum synthesis takes place during the last month of gestation supporting calf health and early immunity. Although research on cow colostrum has focused on individual and environmental factors affecting yield, immunoglobulin levels and physical properties (O’Callaghan et al., 2021; Westhoff et al., 2024), data on how these factors affect the colostrum metabolome are limited. Since colostrum synthesis overlaps with the transition period, its metabolic profile may serve as an indicator of transition programs outcomes.

In this study, we assessed the variability of colostrum metabolome as a function of four feeding strategies from cows that calved during spring, summer and autumn to investigate the effect of transition programs on the physiological status of cows around calving. The combination of univariate and multivariate statistical analyses revealed four and 14 metabolites associated with the feeding strategies and season, respectively. The colostrum metabolites associated with the feeding included fucose, tryptophan, acetic acid and cytidine, whereas those associated with the season included valine, pantothenic acid, isoleucine, caprylic acid, capric acid, lactose, acetoacetic acid, malonic acid, acetylcholine, phosphocholine, creatinine, betaine, aspartic acid and cytidine. The metabolites affected by the feeding strategies are involved in energy metabolism and nitrogen metabolism efficiency in cows. Finally, seasonal patterns were observed in both fatty acids and amino acids. Fatty acid levels increased in summer, while amino acid levels were higher in autumn, showing similar trends previously reported in mature milk.

Colostrum yield and composition, is affected by the prepartum DM and nutrient intake, the parity and season of calving (Westhoff et al., 2024). In the present study, DM and protein intake were significantly decreased only in the FAR-fed cows, which did not affect the colostrum yield (Fig. 2). This is in accordance with previous studies where, different levels of DMI, metabolizable and crude protein intake resulted in similar colostrum yield (Akhtar et al., 2022; Santos et al., 2001; Toghyani & Moharrery, 2015). It has also been reported that colostrum yield is negatively correlated to DM, fat and protein content in colostrum due to dilution effect (Puppel et al., 2019). Although colostrum from FAR-fed cows had significantly lower DM, fat and protein content, no differences were observed in yield. Soufleri et al. (2021) showed that colostrum yield is affected by the calving month and therefore, season. Cows calving during autumn and winter produce less colostrum than those calving in spring and summer. In this study, calving season had no significant effect on colostrum yield. The discrepancies observed may be due to variations in environmental conditions, herd characteristics, management practices across studies or may reflect the fact that newborn calves might have suckled before the first milking affecting the volume of colostrum available for milking.

An important colostrum quality parameter is immunoglobulin (IgG) concentration. Although methods specific to milk IgG exist, colostrum can be tested on-farm using either colostrometers or refractometers, providing a fast and reliable estimation of IgG concentration (Bartier et al., 2015). It has been reported that colostrum with Brix values above 22% is defined as good quality (IgG concentration ≥ 50 mg/mL), while with values < 18% as poor quality colostrum (IgG concentration ≤ 50 mg/mL) (Buczinski & Vandeweerd, 2016). Therefore, it can be concluded that FAR, NH_4_ and OVE samples are of good quality and while MGC samples had the lowest Brix all values exceeded 18%, indicating adequate immunological quality.

Previous studies have shown that altering the prepartum feeding affects colostrum IgG concentration, and therefore Brix (Westhoff et al., 2024). Conneely et al. (2013) and Mann et al. (2016) showed that 1% increase in colostrum DM is associated to increase of 3 g of IgG per colostrum volume. This is in accordance with the trend observed in Fig. 2, where the DM content of MGC samples was significantly lower than that of OVE samples, while FAR, NH_4_ and OVE colostrum samples did not show any significant difference (Fig. 2d), as observed for the Brix (Fig. 2e).

Colostrum metabolome is highly complex and its composition can be influenced by various factors such as the season, diet, cows’ parity and health status therefore, its precise characterization remains challenging (Silva et al., 2024). In this study, 55 metabolites were identified and 47 quantified in colostrum from 89 Danish Holstein cows. All the identified and quantified metabolites in Table 1 have been previously reported in milk (Foroutan et al., 2019), while 32 have been previously identified and quantified in cow’s colostrum (Amarjeet et al., 2025; O’Callaghan et al., 2021). Despite being established metabolites in mature milk, pantothenic acid, ethanol, butyric acid, acetylcholine, acetoacetic acid, glutamine, aspartic acid, dimethyl sulfone, methanol, galactose, cytidine, tyrosine, uracil, tryptophan and histidine were identified and quantified in cow colostrum for the first time.

We previously showed that the FAR, MGC, NH_4_ and OVE feedings have a marginally significant effect (4.5% explained variation, p-value = 0.1) on colostrum metabolome (Tsermoula et al., 2024). It appears that factors beyond feeding influence both the composition of colostrum and the levels of its metabolites. Therefore, a new ASCA model was built, including parity and calving season, showing that both factors significantly affect the colostrum metabolome (p-value = 0.05 and p-value = 0.01, respectively). Although both factors affect mature milk (Grodkowska et al., 2023; Parmar et al., 2020), their impact on colostrum was found to be 2.7% and 7.5% respectively. Since none of the 89 cows were primiparous, the minimal variation due to parity was considered negligible. ASCA results suggest that while the investigated factors influence the colostrum metabolome, its composition is affected by additional factors and possibly dominated by physiological mechanisms.

Univariate analysis of the colostrum metabolome showed that 21 metabolites were significantly affected by the feedings. However, t-test and chi-square analysis showed that other factors influence the variability of most of these metabolites. It was discovered that five factors confound feeding including DMI in the Close-Up and prefresh period, time from calving to colostrum harvest, season and Brix. These findings indicate that associating colostrum metabolites with feeding strategies without accounting for the above confounding factors may result in false positive results and lead to misidentification of metabolites that are partially or solely linked to one or more covariates.

After developing a MLR model and adjusting for the confound factors only fucose, tryptophan, acetic acid and cytidine remained significant for the feeding factor. Fucose is synthesized in the fructose and mannose degradation pathway (Becker & Lowe, 2003) and in sequence, is incorporated into N-glycans found in the membranes, including those of epithelial cells of the mammary gland. N-glycans have a key role in cellular signaling, immune response, and pathogen recognition (Schneider et al., 2017).

During the Close-Up period, it is a common practice to increase dietary starch, while reducing forage and fiber concentrations to increase the DM and energy intake of cows (National academies of sciences engineering and medicine, 2021). In the present study, FAR contained 18 g/Kg starch, while MGC, NH_4_ and OVE feedings 211, 207 and 165 g/Kg starch respectively (Table S1). This resulted in significantly lower NEL20 for the FAR-fed cows than the MGC, NH_4_ and OVE-fed cows (p-value < 0.05; Fig. S1). Under conditions of reduced DM and energy intake, the mammary gland may undergo regression (Capuco et al., 2001), leading to the leakage of cellular components, such as fucose, or the apoptosis of epithelial cells into milk. Although fucose is not directly associated with the metabolic status of cows, it might reflect the status of the mammary gland during early lactation. In our study, corn silage-based feedings, with higher starch concentrations than grass forages, appeared to support cows in coping with energy metabolism around calving.

Amino acids are the building blocks of proteins and play an important role in colostrum metabolism, growth and development of calves (Li et al., 2020). More specifically, tryptophan is an essential amino acid and a precursor to several important compounds, including immunoglobulins, serotonin, and melatonin, which support immune function, reduce stress, and regulate endocrine rhythms (Martinez et al., 2018; Yeste et al., 2020). The concentration of free amino acids in milk, is the result of their increased uptake from the blood stream in the mammary gland and subsequent transfer into milk (Tagari et al., 2004). There are two main sources of tryptophan in blood, the diet (Magan et al., 2019) and intracellular protein degradation (Schlimme et al., 2000).

In the present study, MGC and NH_4_ were identical corn silage-based glucogenic feedings, except for the extra NH_4_Cl added in NH_4_. While FAR and OVE were grass-clover silage ketogenic feeding and a combination of grass diluted MGC and NH_4_ feeding, respectively. Corn is lacking tryptophan (Ernandes et al., 1996), which would explain its low levels in colostrum from corn-fed cows. However, only MGC samples had significantly decreased (p-value = 0.02) tryptophan levels. Ammonium chloride elevates ammonia concentration in cows’ rumen, which is a precursor for microbial crude protein synthesis (Zhu et al., 2022). Microbial protein contains tryptophan that is absorbed in the small intestine (Gresner et al., 2022) therefore, maintaining the tryptophan concentration in the colostrum from NH₄-fed cows at similar levels to the colostrum from FAR and OVE-fed cows.

Acetic acid originates from rumen fermentation and is found in relative high concentrations in peripheral blood, depending on DMI and its uptake by tissues from the bloodstream (Larsen & Kristensen, 2009). Feedings rich in fiber promote the production of acetic acid in the rumen, along with other ketogenic compounds, as opposed to glucogenic diets (van Knegsel et al., 2007). Although the peripheral blood concentration of acetic acid in the present study is not known, the concentration observed in colostrum (0.136 ± 0.045 mM) is lower than its levels in peripheral blood previously reported (1.9 mM) (Raun & Kristensen, 2011), suggesting a concentration gradient consistent with passive diffusion into the mammary gland. Hence, the acetic acid variations observed in colostrum might be explained from the ketogenic or glucogenic nutrient supply to the rumen and their effect on rumen fermentation.

As mentioned above FAR is a ketogenic diet, while MGC and NH_4_ are glucogenic. In OVE feeding, the change from grass diluted corn silage to corn silage feeding may have resulted in delayed adaptation of rumen microbiota (National academies of sciences engineering and medicine, 2021). The increased concentration of acetic acid in colostrum from FAR and OVE-fed cows could be attributed to the ketogenic nutrient supply to the rumen and the lower feed efficiency affecting rumen fermentation, respectively. The involvement of acetic acid in the tricarboxylic acid cycle renders it a marker for monitoring changes in energy metabolism of dairy cows (Enjalbert et al., 2001), suggesting that it is a potential predictor of metabolic status of cows close to calving.

Cytidine is a building block of nucleic acids and main constituent of RNA and is excreted from the lactating cell into the alveolar lumen before secretion to milk (Tiemeyer et al., 1984). The main dietary source of nucleosides, and thus cytidine, is microbial nitrogen, which involves the degradation of dietary nitrogen and synthesis of microbial protein, peptides, free amino acids and nucleic acids (Fujihara & Shem, 2011). Cytidine can also be *de novo* synthesized from liver cells via the pyrimidine biosynthesis pathway. In both cases, cytidine is transferred from the bloodstream into milk across the blood-milk barrier (Schlimme et al., 2000). It has been proposed that cytidine could be used as an indicator for the efficiency of nitrogen use from dairy cows (Stentoft et al., 2014). In the present study, FAR feeding had grass silage as the main nitrogen source, while MGC, NH_4_, and OVE canola cake, indicating that grass silage was more beneficial in the microbial synthesis of cytidine. However, further research is needed to validate this finding and to elucidate the underlying mechanisms.

A previous study showed that season affects mature milk metabolome, especially when lactating cows are pasture-fed (Zhu et al., 2021). In this study, feedings were controlled, excluding variations coming from the botanical and chemical compositions of fresh forages. Nevertheless, seasonal temperature differences affect milk composition, in particular fatty acids. During summer, saturated fatty acid levels decrease, whereas monounsaturated, polyunsaturated, and conjugated linoleic acids increase in mature milk. (Collomb et al., 2008). Milk collected in autumn has the highest concentration of phospholipids (Liu et al., 2017). Although the total saturated fatty acids were not measured in this study, capric and caprylic acid levels significantly increased in summer. Seasonality has been also related to free amino acid levels in milk. In a previous study, serine and glutamic acid increased from spring to autumn (Cabrera et al., 2024), as observed for valine and isoleucine in the present study.

Colostrum quality tends to be better in cooler seasons, with increased levels of fat, protein, and immunoglobulins, likely due to higher DMI and reduced heat stress (Westhoff et al., 2024). In the present study, most of the metabolites increased during spring and only pantothenic acid, valine and isoleucine were found at increased levels in autumn. The limited knowledge on the metabolic and endocrine signals that regulate colostrum synthesis along with the lack of data on environmental conditions in this study, restrict our ability to explain these differences in the colostrum metabolome.

To the best of authors’ knowledge, this is the first study to investigate associations between cow colostrum metabolites and feeding strategies in transition period across seasons. The present findings show that factors beyond feeding strategies, season of calving and individual animal factors influence colostrum synthesis. However, this study has certain limitations. Firstly, information on environmental conditions was not recorded, which hindered us from including them in the evaluation of the colostrum metabolome across seasons. In addition, the inclusion of primiparous cows would allow the evaluation of parity on colostrum metabolome. Further research is required to evaluate our findings and the mechanisms underlying the shift of fucose, tryptophan, acetic acid and cytidine levels in colostrum. Finally, studies that isolate and manipulate environmental conditions, including temperature, day length and humidity, independently under controlled settings are needed to establish causality and provide the foundation for explaining the seasonal variability of cow colostrum metabolome.

## Conclusions

Although colostrum metabolome is influenced by various factors, the observed changes in this study are relatively small in magnitude. Despite the limited impact of the feeding strategies and season on the colostrum metabolome, specific metabolites have the potential to serve as markers of the cow metabolic status around calving. Acetic acid is a candidate of cow energy metabolism; cytidine may serve as an indicator of nitrogen metabolism efficiency, while fucose and tryptophan could provide insights into the effect of metabolic stress on mammary gland and non-protein nitrogen supplementation, respectively.

According to the authors’ knowledge, this is the first study showing a shift in the regulated colostrum metabolome due to a change in prepartum feeding. Although these metabolites show promising potential, further research is required to validate them and understand the underlying mechanisms. This study supports that colostrum synthesis and excretion are primarily regulated by intrinsic physiological mechanisms. Essentially, maternal metabolism before parturition adjusts to optimize colostrum production; therefore, combining urine metabolomics with colostrum metabolomics may provide a better overview of the metabolic status of cows around calving.

## Supplementary Information

Below is the link to the electronic supplementary material.

Supplementary Material 1—Table S1. Study design and composition of feeding from the feeding trial of the 89 cows. Figure S1. Box plots with dry matter, energy and protein intake of cows. Table S2. Colostrum metabolites with significantly different levels across seasons (word).

## Data Availability

The data analyzed during the current study are available from the corresponding authors on reasonable request.
