# Supplementary material for: Pre-partum feeding strategies affect colostrum metabolite levels related to nitrogen and energy metabolism in Holstein dairy cows
Source: Metabolomics. 2025 Aug 29;21(5):128. doi: 10.1007/s11306-025-02329-w (PMC12397122; doi:10.1007/s11306-025-02329-w)
Supplement: Supplementary file 1 — Supplementary Material 1—Table S1. Study design and composition of feeding from the feeding trial of the 89 cows. Figure S1. Box plots with dry matter, energy and protein intake of cows. Table S2. Colostrum metabolites with significantly different levels across seasons (word). [file 11306_2025_2329_MOESM1_ESM.docx]

**Supplementary material**

Pre-partum feeding strategies affect colostrum metabolite levels related to nitrogen and energy metabolism in Holstein dairy cows.

Paraskevi Tsermoula^a*^, Niels Bastian Christensen^b¥^, Bekzod Khakimov^a*^

^a^Department of Food Science, University of Copenhagen, Rolighedsvej 26, Frederiksberg, 1958, Denmark

^b^SEGES Innovation P/S, Agro Food Park 15, DK 8200 Aarhus N, Denmark

^¥^Current address: Department of Animal and Veterinary Sciences, Aarhus University Viborg, AU-Foulum, 8830 Tjele, Denmark

Table of contents

Table S1. Study design and composition of feeding from the feeding trial of the 89 cows

Figure S1. Box plots with the dry matter, energy and protein intake of cows

Table S2. Colostrum metabolites with significantly different levels across seasons

**Study design**

Ninety-nine cows were randomly allocated to four experimental feeding treatments during the dry period. The four different treatments are:

1. FAR: feeding of the grass based Far-OFF dry ration from dry off to calving.
2. MGC: feeding a grass based Far-OFF ration from dry off until three weeks before expected calving and feeding a corn silage based Close-UP ration acidified with magnesium chloride until calving.
3. NH_4_: feeding a grass based Far-OFF ration from dry off until three weeks before expected calving and feeding the Close-UP ration with added ammonium chloride until calving.
4. OVE: feeding a grass based Far-OFF ration from dry off until three weeks before expected calving, feeding a grass diluted (20:80, on dry matter basis), MGC ration the first week of the Close-UP period and thereafter feeding the NH_4_ Close-UP ration until calving.

The Far-OFF ration was based on grass silage and barley straw and it was formulated for an organic matter digestibility of 65 %. Soybean meal was included in the Far-OFF ration to maintain the crude protein concentration of at least 110 g/kg on dry matter basis. The Close-UP- MGC ration was based on corn silage and canola cake. The ration was formulated for a starch concentration of 200 g/kg on dry matter basis and minimum crude protein concentration of 130 g/kg. The starch concentration was balanced by substituting sugar beet pulp for corn silage. All rations were weekly based on three weeks average measurements on silage composition. The ingredient and nutrient composition of the treatments are detailed in (Table S1). After calving all cows received a common lactation TMR (Total Mixed Ration), optimized using the NorFor feed evaluation system for lactating cows (Volden et al., 2011).

**Table S1**. Ingredient (% of dry matter) and nutrient composition (g/kg if not otherwise stated) of experimental diets.

|  | Far-OFF | Close-UP - MGC | Close-UP – NH_4_ | Close-UP - OVE | |
| --- | --- | --- | --- | --- | --- |
| Corn silage |  | 66.1 – 71.2 | 63.5 – 68.4 | 52.9 – 57.0 | |
| Grass-clover silage | 51.9 – 68.1 |  |  | 20.0 | |
| Barley straw | 27.2 – 42.0 |  |  |  | |
| Canola cake |  | 23.1 | 22.2 | 18.5 | |
| Soybean meal | 0 – 5.3 |  |  |  | |
| Sugar beet pulp |  | 3.7 – 8.9 | 3.6 – 8.5 | 3.0 – 7.1 | |
| Mineral premix Type-3* | 0.8 |  |  |  | |
| Mineral premix Close-UP** |  | 0.4 | 0.4 | 0.3 | |
| Magnesium chloride hexhydrate |  | 1.5 – 1.8 | 1.4 – 1.7 | 1.2 – 1.4 | |
| Ammonium chloride premix*** |  |  | 0,4 |  | |
| Hygiene4Feed**** | 0 – 0.1 | 0 – 0.1 | 0 – 0.1 | 0 – 0.1 | |
| Nutrients | | | | |  |
| DM (g/kg) | 378 | 392 | 395 | 375 | |
| Ash (g/kg) | 83 | 63 | 66 | 71 | |
| Crude protein (g/kg) | 118 | 135 | 137 | 138 | |
| OMD, % | 68 | 78 | 78 | 78 | |
| Crude fat (g/kg) | 25 | 47 | 47 | 45 | |
| NDF (g/kg) | 543 | 342 | 337 | 369 | |
| Starch (g/kg) | 18 | 211 | 207 | 165 | |
| Sugar (g/kg) | 38 | 22 | 23 | 27 | |
| AAT20^¥^ (g/kg) | 70 | 91 | 91 | 87 | |
| NEL20 (g/kg) | 47 | 64 | 64 | 62 | |
| Mineral composition^†^ | | | | |  |
| Ca (g/kg) | 8,2 | 4,7 | 4,7 | 5,7 | |
| P (g/kg) | 3,2 | 4,5 | 4,3 | 4,5 | |
| Mg (g/kg) | 3,1 | 4,0 | 4,0 | 3,9 | |
| Na (g/kg) | 2,3 | 1,5 | 1,8 | 1,6 | |
| K (g/kg) | 28,3 | 11,6 | 10,6 | 14,5 | |
| S (g/kg) | 2,4 | 2,6 | 2,6 | 2,8 | |
| Mn (g/kg) | 105 | 61 | 63 | 68 | |
| Zn (g/kg) | 77 | 68 | 72 | 68 | |
| Cu (g/kg) | 18 | 9 | 9 | 9 | |
| Cl (g/kg) | 13,4 | 8,5 | 10,2 | 9,6 | |
| DCAD^¥^ (meq./kg) | 299 | -39 | -96 | -4 | |

*Mineral premix Type-3, ViloFOSS, Gråsten, Denmark. Composition (%): calcium carbonate, 36.9; sodium chloride, 30.1; magnesium oxide, 25.7; molasses, 2.9; calcium magnesium carbonate, 1. Analytical composition (%): Ca, 14.6; Mg, 14.1; Na, 11.6. Additives (per kg): Vitamin A, 600.000 IE; Vitamin D3, 190.000; Vitamin E, 4.000 IE; Cu, 1.500 mg; Mn, 4.000 mg; Zn, 4.500 mg, I, 225 mg; Se, 50 mg; Co, 25 mg.

**Vilomin 9941850 close up basis, Vilomix, Mørke, Denmark. Composition (% of DM): sodium chloride 66.7; wheat 10. Analytical composition (%): Ca, 0.1; Na, 25.9; Mg, 0.1; Crude ash, 80.7. Additives (per kg): Vitamin A, 416.700 IE; Vitamin D3, 416.700; Vitamin E, 55.883 IE; Cu, 833 mg; Mn, 5.000 mg; Zn, 5.000 mg, I, 17 mg; Se, 42 mg; Co, 66.7 mg.

***Vilomin 9941851 Ammon NaCl, Vilomix, Mørke, Denmark. Composition (% of DM): sodium chloride 20.0; wheat 15.0 Analytical composition (%): Ca, 0.1; P, 0.1; Na, 7.8; Mg, 0.1; Crude ash, 33.3. Additives (per kg): ammonium chloride, 500.000 mg.

**** Hygiene4Feed (Biochem Zusatzstoffe Handels- und Produktionsgesellschaft mbH, Lohne, Germany). Additives (per kg): Potassium sorbate, 955.000 mg.

^¥^AAT20: amino acids absorbed in the small intestine at 20 kg DMI/day

**Figure S1.** Box plots showing daily intake of dry matter (DMI), net energy for lactation (NEL20) and crude protein (cp) during the Close-Up and prefresh period for the cows as allocated to the FAR, MGC, NH_4_ and OVE feedings.


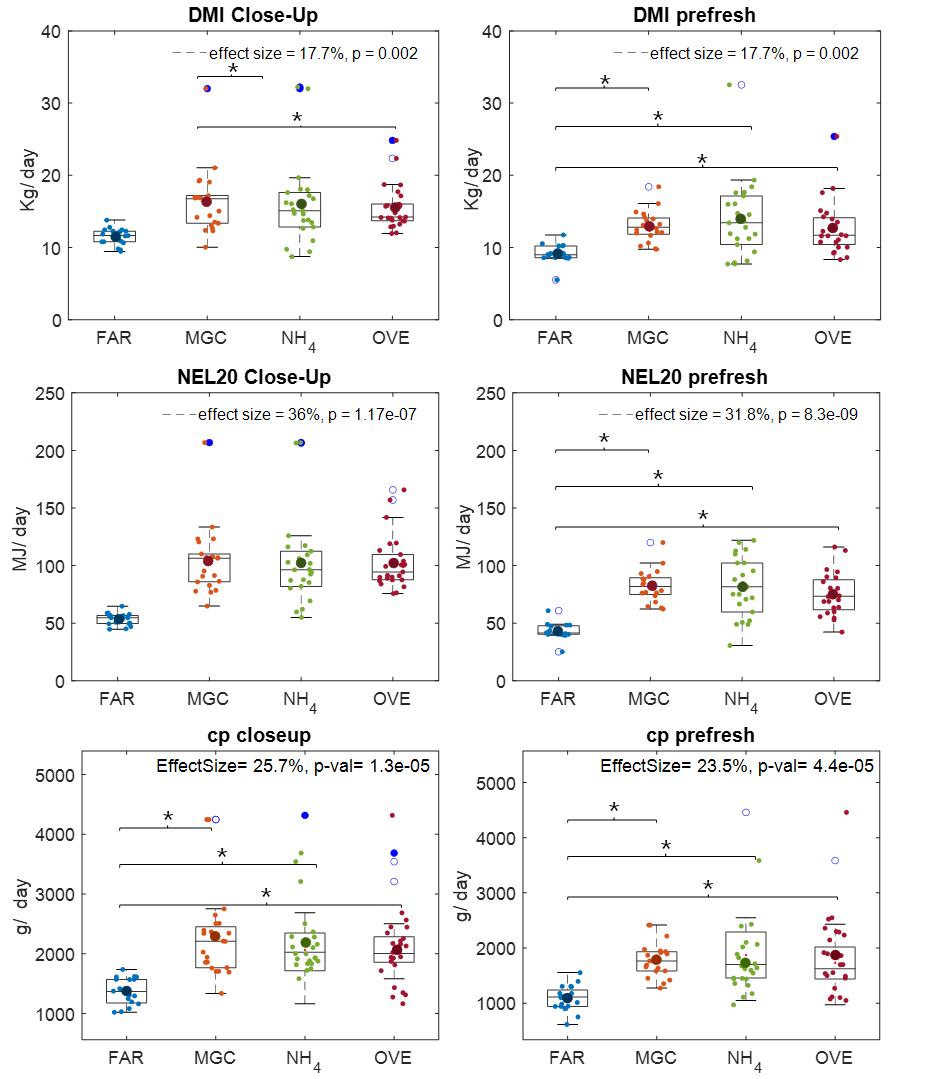


**Table S1.** List of colostrum metabolites found to be different between the spring, summer and autumn seasons by ANOVA. p-values < 0.05 and 0.001 are considered significant, while (-) are considered non-significant.

|  | **p-value** | **effect size (%)** | **p-value** | | | | |
| --- | --- | --- | --- | --- | --- | --- | --- |
|  |  |  | **spring-summer** | **summer-autumn** | | **spring-autumn** | |
| pantothenate | 0.007 | 11.6 | - | | 0.01 | | 0.02 |
| acetoacetate | < 0.001 | 39.0 | 0.01 | | 2.1E-05 | | 4.5E-09 |
| lactose | 0.005 | 12.0 | - | | 0.006 | | 0.04 |
| valine | 0.007 | 11.6 | - | | 0.03 | | 0.009 |
| bin (caprylic acid + capric acid) | 0.01 | 10.5 | 0.03 | | 0.04 | | - |
| malonate | 0.02 | 9.02 | - | | - | | 0.02 |
| bin (acetylcholine + phosphocholine) | 0.006 | 11.9 | - | | - | | 0.004 |
| isoleucine | 0.04 | 7.58 | - | | - | | 0.04 |
| cytidine | 0.04 | 7.34 | 0.03 | | - | | - |
| creatinine | 0.02 | 9.51 | - | | - | | 0.02 |
| aspartate | 0.04 | 7.40 | 0.03 | | - | | - |
| betaine | 0.02 | 8.69 | 0.03 | | - | | 0.05 |
